# All People Are Winners

**DOI:** 10.1007/978-3-030-47408-9_5

**Published:** 2020-07-01

**Authors:** Max Meyer

**Affiliations:** Bern, Switzerland

## Abstract

Up until the eighteenth century, most people in Europe lived in severe poverty. They had no running water, no sewage systems, no medical care and hardly enough to eat. Liberalism has brought people freedoms and levels of prosperity that have never before existed. But it’s not only the people living in developed first-world countries who are enjoying a level of prosperity never before experienced by mankind. The standard of living has risen everywhere else as well. Most people (80%) can now meet their basic needs. There are the very wealthy regions (Europe, North America, Japan, South Korea, Taiwan, Singapore); and there are extremely poor regions (about 9%) with unacceptable living conditions.

## Prosperity Is Increasing Worldwide

Liberalism has brought people freedoms and levels of prosperity that have never before existed. This is true both for rich Western and Asian countries and countries that were previously poor. It is a long-term positive development that has always had to deal with setbacks.

Up until the eighteenth century, most people in Europe lived in severe poverty. They had no running water, no sewage systems, no medical care and hardly enough to eat. Many died of starvation, disease, or epidemics. Only the very rich—the aristocrats—did reasonably well. At that time, people in Europe lived no differently than people in the poorest countries in the world do today.

But it’s not only the people living in developed first-world countries who are enjoying a level of prosperity never before experienced by mankind. The standard of living has risen everywhere else as well. Only in a few of the poorest countries are there people still dying of starvation and according to the UN Agenda 2030 from 2015,^1^ extreme poverty should be eradicated within a few years. This forecast states that 380 million Africans will still be among the poorest, while elsewhere around 50 million people will continue to be affected by poverty. This is less than 6% of the world’s population. Asians in particular will be able to escape poverty, while in Central Africa (and, depending on armed conflicts, in Middle Eastern countries) the number of people living in extreme poverty will increase. In these regions there are far too many refugees who are fleeing wars, economic crises, policy failures and—by extension—poverty and hunger.

In all other “poor” countries there is no longer any starvation.^2^ In these countries, 60% of all girls attend school. 88% of the children have been vaccinated and 85% of the people have electricity. In general, it can be said that about 80–90% of the world’s population can meet their basic needs. Therefore, real progress has been made all over the world.

## Previously-Poor Regions Also Benefit

Time and again, it has been claimed that capitalism increases prosperity in developed countries while exploiting developing countries. However, as a quick look at the globe shows, developing countries that have a market economy combined with good governance (Chap. 10.1007/978-3-030-47408-9_11) stand a genuine chance of joining the ranks of the successful nations. Japan, Singapore, South Korea, and Taiwan have all achieved this within one or two generations. Many so-called emerging markets such as India and Brazil are well on their way to doing so. The same holds true for China if they change their systems and introduce good governance. Others believe they have been left behind. They speak of a first world elite who have mastered new technologies and are driving them forward, increasing the distance to those left behind and leaving them no opportunity to catch up. They fail to recognize the culpability of members of their own corrupt governments, who enrich themselves rather than invest in infrastructure and good governance.

Thousands of years ago, mankind began at the lowest level of prosperity. Gradually, the situation improved. However, even in the eighteenth century, many Europeans went to bed hungry and it took generations for prosperity to come. Poor countries are making progress more quickly today and it takes only a few generations to move from one level of prosperity to the next.

According to the IMF, the poorest countries have the highest rates of growth (2–6%—sometimes even higher). Wealthy countries are growing considerably more slowly (2–4%). Eventually, the poor countries will catch up. Provided they have developed a liberal democracy, income levels will equalize. What will happen then? People from previously poor countries are used to hard work and these countries will overtake the rich ones—until they have gotten used to prosperity (for example, Japan). Here too, development comes in waves. Many ambitious Europeans have already noticed that they have more opportunity in Asia than in Europe because growth rates are better. As a result, they are trying their luck there.

In addition:

- Average life expectancy is continuously increasing. In 1900, men born in Germany lived an average of 46.4 years and women 52.5 years. Today men are expected to live 78.4 years and women 83.2 years. For most people on the planet, the average life expectancy has risen sharply.^3^
- When considered over the long-term, the difference between rich and poor is decreasing. In the past it was much more extreme. Until the eighteenth century, the rich had sovereignty over the poor rural population who were tied to the land and held as slaves. Since then, the difference between the two has continuously been reduced and statistics show that in most countries this continues to be the case.^4^ Of course, there have also been setbacks, but the long-term development is clear and very positive.
- Crime, when considered from a long-term perspective, is decreasing.^5^
- War deaths are also declining, despite possible impressions to the contrary from the Middle East.
- Attitudes to war have changed considerably. In ancient Greece, death in battle was necessary to gain access to the afterlife. Napoleon was considered a hero, whereas Hitler was a criminal. During World War I, soldiers went enthusiastically to war, writing “On to Paris” on the railway wagons that carried them. They spoke of “Fields of Honor”. Today if there is war, it is considered a crime.
- Epidemics are decreasing or they rarely occur anymore. In 1918, the Spanish Flu killed 2.7% of the world population. At the beginning of 2009, the Swine Flu lasted 2 weeks killing 31 people. Action taken thanks to global cooperation, modern medicine and technology will hopefully limit fatalities from the coronavirus.
- Since 1990, child mortality rates among children under the age of five have decreased by more than half, from 12.5 million children to 5.3 million in 2018. More than 80 countries, including many of the poorest, have been able to reduce child mortality rates by two thirds since 1990. (UNICEF Report, September 2019)

As Hans Rosling points out in his book, *FACTFULNESS*, many people do not see the progress that has been made. They lack a *fact-based* perspective. This could be because they are using outdated information; they are not familiar with the correct facts; or because they ignore facts that do not agree with their world view. Here is an example of outdated information from the late 1990s: In 1997, 42% of the world’s population lived in extreme poverty. In 2019 only 9% did. In the last 20 years the number of people living in extreme poverty has decreased by half. Although the majority of people do not enjoy the same standard of living as the middle class in wealthy countries, they have enough to eat, their young girls can attend school, and their children receive basic medical care and are vaccinated. Step by step, conditions in the world are improving.

In summary, most people (80%) can now meet their basic needs. There are the very wealthy regions (Europe, North America, Japan, South Korea, Taiwan, Singapore); and there are extremely poor regions (about 9%) with unacceptable living conditions.^6^

Everything has improved!—What counts are the facts and not personal perception. Liberalism has increased prosperity almost everywhere in the world.

## Footnotes

1 Resolution of the UN General Assembly adopted on 25 September 2015 Nr. 70/1. “Transforming our world: The Agenda 2030 for sustainable development”. https://www.un.org/Depts/german/gv-70/band1/ar70001.pdf

2 The figures in this chapter are based on the highly recommended book, *FACTFULNESS (Ten reasons we’re wrong about the world and why things are better than you think)* by Hans Rosling.

3 Life expectancy has more than doubled since the 1870s. Here’s a detailed link: https://de.statista.com/statistik/daten/studie/185394/umfrage/entwicklung-der-lebenserwartung-nach-geschlecht/. During the Middle Ages, the average life expectancy for women was 25 years and for men 32 years. There is more information here: https://www.google.ch/search?q=Lebenserwartung+historisch&sa=X&ved=2ahUKEwjw946QrKHmAhVjwqYKHcnzAcoQ3rMBKAJ6BAgNEAk&biw=1745&bih=850

4 Source: The statistics for “Standard of Living and Inequality of Income Distribution in Selected European Countries” from the Swiss Federal Office of Statistics, which refers to Eurostat—EU-SILC 2015 (Version from March 28, 2017), or the article from the Neu Zuericher Zeitung “Income Differences are Decreasing”. (https://situation-bevoelkerung/soziale-situation-wohlbefinden-und-armut/ungleichheit-der-einkommensverteilung.html).

5 Source: Swiss Police Crime Statistics https://www.bfs.admin.ch/bfs/de/home/aktuell/neue-veroeffentlichungen.assetdetail.7726202.html

6 The largest nutritional problem in the world today is not malnutrition but overnutrition, the urge to overeat by consuming fat and sugar resulting in obesity. We must teach children to avoid sweets and chips because there are too many of these and because of the enormous problem of food waste.

